# Calcium Blood Level Elevation After Atorvastatin Initiation in a Patient With Hyperparathyroidism

**DOI:** 10.7759/cureus.53306

**Published:** 2024-01-31

**Authors:** Michael Rechter, Michael Hauzer

**Affiliations:** 1 Internal Medicine, Meir Medical Center, Kfar Saba, ISR; 2 Family Medicine, Clalit Health Services, Haifa, ISR

**Keywords:** drug-related adverse event, atorvastatin, prevention in primary care, hyperparathyroidism, hypercalcemia

## Abstract

Atorvastatin is a very common medication used for lowering blood cholesterol levels. The drug has known adverse effects, but an elevation in calcium levels is not listed as one of them. We report a 52-year-old man with hyperparathyroidism and hypercholesterolemia, who, under treatment with atorvastatin, developed an additional rise in calcium levels. He was asymptomatic, and during the investigation of his hypercalcemia, a drug adverse effect was suspected. Therefore, atorvastatin therapy was stopped, and calcium levels dropped as a result. Subsequent readministration of atorvastatin and its cessation produced similar results. While hypercalcemia is not listed as a common adverse effect of atorvastatin, we introduce such a phenomenon along with possible underlying mechanisms. Although our patient was asymptomatic, hypercalcemia can be a dangerous condition, especially in a population where the initial calcium levels are already elevated.

## Introduction

Hypercalcemia, characterized by abnormally high serum calcium levels, presents a spectrum of clinical manifestations ranging from asymptomatic to life-threatening complications. Normal calcium values range between 8.5 and 10.5 mg/dL, while values between 10.5 and 12 mg/dL, 12.1 and 14, and above 14 are considered mild, moderate, and severe hypercalcemia, respectively. Most patients with hypercalcemia are asymptomatic, it can escalate into a life-threatening condition. The common etiology for hypercalcemia differs depending on the medical setting. Primary hyperparathyroidism is considered the most common etiology for hypercalcemia in outpatients, while hypercalcemia associated with malignancy is the most common among hospitalized patients. Hypercalcemia can be caused by various conditions, such as granulomatous diseases, genetic disorders (e.g., familial hypocalciuric hypercalcemia), hyperthyroidism, and adrenal insufficiency [1]. Certain medications, such as vitamin D and vitamin A supplements, thiazide diuretics, lithium, and calcium-containing antacids, can also induce hypercalcemia [2].

Statin medications are frequently prescribed to reduce cholesterol levels. They are among the most widely used medications, with common adverse effects including myalgia, elevation of liver transaminases, interaction with other medications, and more [3]. However, a case report by Ipekçi et al. has associated statin use with hypercalcemia. The proposed mechanisms for hypercalcemia were altered vitamin D levels, increased bone resorption, drug interaction, and idiosyncratic reactions [4].

Research findings on the effect of statins on vitamin D remain equivocal. While certain studies report elevations in vitamin D, others indicate reductions. A hypothesis regarding how statins elevate vitamin D suggests that by inhibiting HMG-CoA reductase, statins lead to the accumulation of 7-hydrocholesterol, a precursor of 25-OH vitamin D. The increased availability of this precursor could potentially contribute to elevated vitamin D levels and subsequent intestinal calcium absorption [5].

Concerning increased bone turnover, statins are usually considered as medications that promote osteogenesis, signifying the opposite effect [6]. According to a review by Chamani et al., statins influence different mediators of bone growth, such as bone morphogenetic protein 2, type I collagen, glucocorticoids, and others. Importantly, the authors emphasize that the available data on the effects of statins on bone osteogenesis do not originate from well-planned randomized controlled trials (RCT). They suggest that future research should further investigate the role of statins in bone diseases [7].

In terms of drug interactions, atorvastatin undergoes metabolism in the intestine and liver, and approximately 85% of its liver metabolism is mediated by CYP3A4. Therefore, inhibitors of CYP3A4, such as antifungals, macrolide antibiotics, calcium channel antagonists, etc., can increase atorvastatin plasma concentration. Furthermore, recent observations suggest potential drug interactions that could inhibit the hepatic uptake of atorvastatin via OATP1B transporters, consequently leading to an increase in its plasma levels [8]. Regarding idiosyncratic reactions, in rare cases, an individual’s susceptibility could play a role in statin-induced hypercalcemia, potentially involving mechanisms not yet understood. Although hypercalcemia is not reported as a common adverse effect, our case exemplifies an atorvastatin-induced hypercalcemia exacerbation. The patient provided informed consent for publishing this case report.

## Case presentation

**Figure 1:**
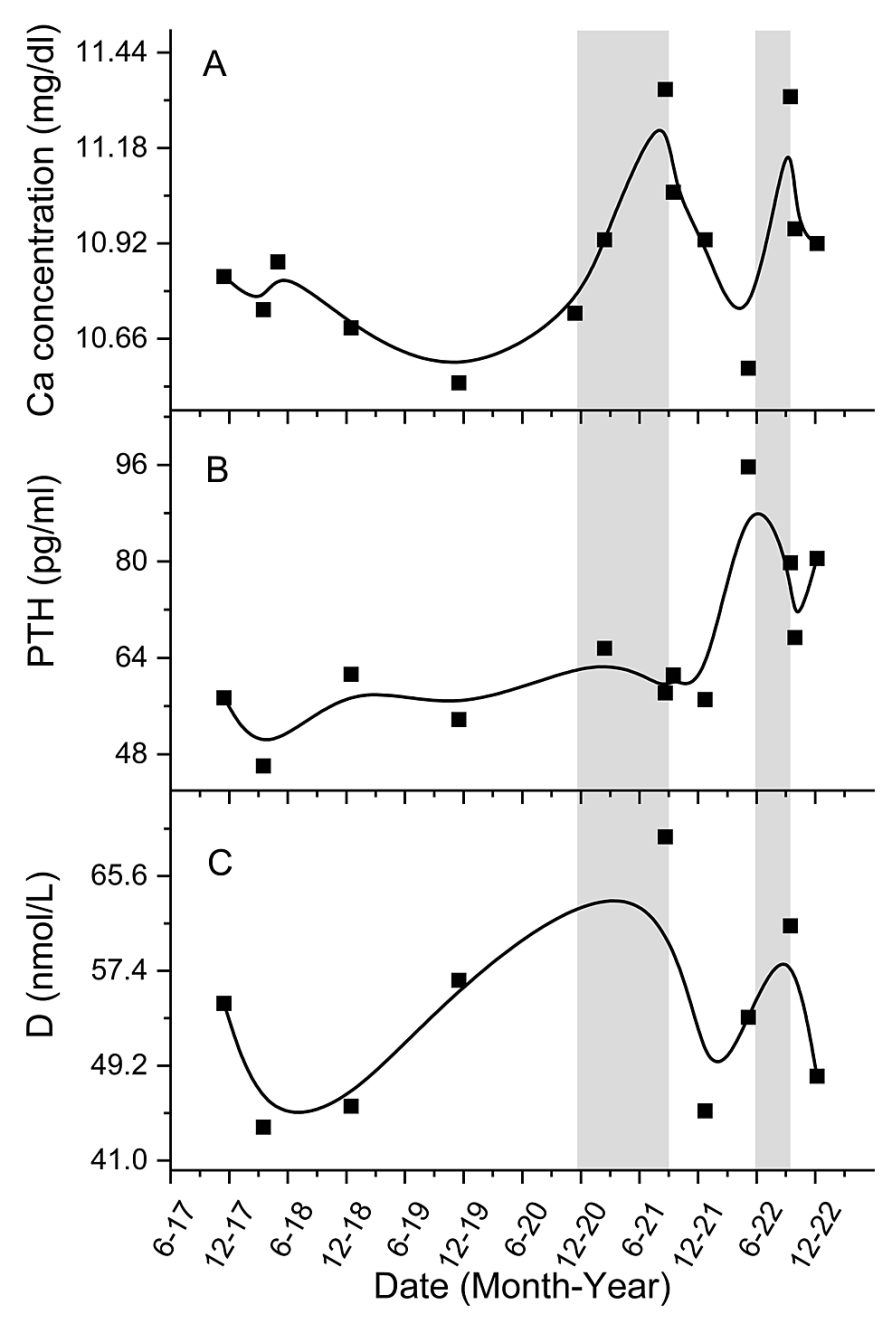
Blood concentration of calcium, parathyroid hormone (PTH), and vitamin D over five years, B-spline fit. (A) Calcium concentration; (B) PTH concentration; (C) 25-OH vitamin D concentration. The gray area represents the time atorvastatin was used (atorvastatin administration started in November 2020, stopped in September 2021, renewed in June 2022, and finally stopped in September 2022).

We present a case of a 52-year-old man who experienced an exacerbation of hypercalcemia discovered during a routine follow-up in the clinic. His medical history was significant for hyperparathyroidism and hypercholesterolemia. Periodic blood tests for calcium revealed stable levels, maintaining around 10.8 mg/dL since 2017 (Figure 1A exhibits his calcium concentration over time). From the beginning of 2021, he experienced a rise in his calcium levels reaching 11.34 mg/dL, while remaining asymptomatic.

When hypercalcemia was initially observed, he underwent various tests including a 24-hour urine collection conducted in 2017 to assess calcium and phosphorus levels, which yielded results within the normal range. Similarly, protein electrophoresis performed in 2018 exhibited normal findings. A rheumatic panel conducted in the same year showed no pathological indications. Regarding the diagnosis of hyperparathyroidism, a four-dimensional computerized tomography (CT) scan performed in March 2020 raised suspicion of a parathyroid adenoma located under the left lower border of the thyroid gland.

In November 2020, because of hypercholesterolemia and a calculated 10-year risk of 3.36% to an atherosclerotic cardiovascular disease according to the SCORE of the European Society of Cardiology, treatment with atorvastatin 20 mg was started as a primary prevention after a shared decision-making conversation. He had no other comorbidities and received no other medications including multivitamins or vitamin D supplements.

When evaluating the patient during the additional recent rise in calcium levels, the physical exam was unremarkable. All blood tests were in the normal range (including blood count, liver enzymes, total protein, globulin, albumin, electrolytes, kidney function, and TSH, except lipids). Notably, we have documentation of his blood tests since November 2017, as detailed in Table 1. Looking at additional tests for a broader period, it is evident that his phosphorus was usually low (ranged between 2.08 and 2.83 mg/dL), his parathyroid hormone (PTH) was usually high (ranged between 44.4 and 95.7 pg/mL) (Figure 1B), and his 25-OH vitamin D was considered mildly low (ranged between 43 and 69 nmol/L) (Figure 1C). Unfortunately, 1,25-OH vitamin D is not part of the regular follow-up in the community setting and, hence, was not available. Additionally, as part of age-appropriate screening for malignancy, which can cause or exacerbate hypercalcemia, a colonoscopy conducted in December 2021 was assessed as normal.

**Table 1:** Blood concentration of calcium, parathyroid hormone (PTH), vitamin D, albumin, and phosphate over five years. Abnormal values are bolded.

Test (normal range)/date (D/M/Y)	Calcium (8.5-10.5 mg/dl)	PTH (10-58 pg/ml)	Vitamin D (76-125 nmol/L)	Albumin (3.5-5.2 g/dL)	Phosphate (2.5-5 mg/dl)
19/11/2017	10.83	57.4	54.6	4.59	2.32
22/03/2018	10.74	46.1	43.9	4.7	2.53
06/05/2018	10.87			5.03	2.83
20/12/2018	10.69	61.3	45.7	5.03	2.62
21/11/2019	10.54	53.8	56.6	4.64	2.08
17/11/2020	10.73			4.64	2.57
17/02/2021	10.93	65.6		4.64	2.6
25/08/2021	11.34	58.2	69	4.64	2.81
19/09/2021	11.06	61.2		4.6	2.3
27/12/2021	10.93	57.1	45.3	4.78	2.38
11/05/2022	10.58	95.7	53.4	4.66	2.32
19/09/2022	11.32	79.8	61.3	4.66	2.35
03/10/2022	10.96	67.4		4.44	
11/12/2022	10.92	80.5	48.3	4.62	2.45

To find the cause of his elevated calcium levels, different etiologies were sought. One of those was an aggravation of his hyperparathyroidism, which was usually well-balanced. Looking at his PTH levels in Figure 1B, it is evident they did not change after receiving atorvastatin, thus weakening this hypothesis. Because globulin and protein were normal, multiple myeloma was not suspected. Hyperthyroidism can also cause hypercalcemia, but his TSH levels were in the normal range. TSH levels were measured two months before atorvastatin was started and three months after stopping the medication.

The only change in his lifestyle was the addition of atorvastatin 20 mg in November 2020. As a drug's adverse effect should always be suspected, atorvastatin usage might have caused this condition. Atorvastatin was discontinued in September 2021 and switched to ezetimibe 10 mg daily. He was encouraged to continue a close follow-up in the clinic over the following weeks and to perform additional blood tests to observe calcium levels. If calcium levels had not been affected, other steps would have been taken, such as performing additional tests and referring the patient to an endocrinologist.

On follow-up, his calcium improved to 10.58 mg/dL. Figure 1A shows the rise and fall of calcium levels after receiving and stopping atorvastatin, respectively. To strengthen a causal relationship between atorvastatin and elevated calcium levels, a second administration of atorvastatin 20 mg was started in June 2022, with the patient’s informed consent. In response, his calcium levels increased again to 11.32 mg/dL by September 2022. Atorvastatin was discontinued followed by a decrease in calcium levels once more.

## Discussion

This case suggests atorvastatin can exacerbate hypercalcemia in a patient with underlying hyperparathyroidism. The rises and falls in calcium levels observed during the two administrations and cessations of atorvastatin, respectively, suggest potential evidence of a causative effect. As previously mentioned, Ipekçi et al. described a similar case. In their report, they documented a 54-year-old female who was treated with atorvastatin and was admitted to the hospital because of mild hypercalcemia. Despite an extensive inquiry, there was no apparent reason for her hypercalcemia other than statin treatment. Atorvastatin was discontinued, and calcium levels returned to normal. The readministration of atorvastatin and stopping produced similar results. The mechanism of atorvastatin-induced hypercalcemia is unclear. Ipekçi et al. proposed several possible mechanisms including bone reabsorption, increased vitamin D concentrations, drug interaction, and an idiosyncratic reaction [4]. In our case, an exacerbation of hyperparathyroidism should also be suspected.

A study performed by Hernández et al. in 2012 compared 478 statin users to 1837 non-statin users, with 84% of statin users having been on the medication for more than one year. Contrary to our observation, they showed no significant difference in the blood levels of calcium, 25-OH vitamin D, and PTH when comparing the two groups. However, it is important to note that among the statin users, only 30% were taking atorvastatin. In terms of bone metabolism, there was a significant decrease in bone turnover markers in the statin treatment group, indicating the favorable effect of statin on bone turnover [9].

Ipekçi et al. observed an elevation in the deoxypyridinoline/creatinine while the patient was taking atorvastatin, indicating heightened osteoclastic activity. Furthermore, the ratio returned to normal upon discontinuation of the medication [4]. Despite this observation and the lack of well-designed RCTs, most studies support the anabolic effect of statin on bones, thereby challenging the theory of bone turnover as a mechanism for hypercalcemia [6,7].

Relative to the vitamin D-associated mechanism, conflicting data exist regarding the impact of statins on vitamin D. This contradiction can be observed in a meta-analysis from 2017 that compared seven studies, three of which were RCTs. The RCTs showed a significant increase in vitamin D levels of 2.71 ng/mL when treated with statins. Contrarily, non-RCT studies showed a significant decline in vitamin D levels of -0.70 ng/mL when treated with statins. The authors concluded that additional studies should be conducted to examine the favorable effect of the RCTs [5]. A large sample size study of 6261 patients performed by Orces et al. in 2020 offered additional information on this conflictual subject. They showed that vitamin D was significantly higher among atorvastatin, rosuvastatin, and simvastatin users compared to non-statin users [10].

Although Orces et al. researched patients above the age of 69.5, we believe that the same principle should apply to our patients [10]. Considering the B-spline fit curve in Figures 1A, 1C, it seems that calcium and vitamin D levels are in alignment. After starting atorvastatin, both climbed and when stopping atorvastatin for the first time, both declined simultaneously. This observation, among the claims that atorvastatin raises vitamin D levels, can support the theory that vitamin D is responsible for atorvastatin-induced hypercalcemia.

Another possible explanation for the rise of calcium blood levels is the PTH-mediated mechanism when starting atorvastatin PTH levels have not changed. Afterward, when stopping and renewing the medication, PTH and calcium move in opposite directions, as seen in Figures 1A, 1B. If PTH was responsible for the additional rise in calcium levels, we would expect a parallel pattern as an increase in PTH levels would cause an increase in calcium levels as well. For that reason, it seems that according to our data, PTH is less likely to be the cause of atorvastatin-induced hypercalcemia, and it simply reacts to calcium blood levels. As for drug interaction, the patient was not receiving any other medication, including vitamin D supplementation; hence, this explanation is not likely in our case.

Among the strengths of this study are the additional tests besides calcium that are available over a long period, such as vitamin D and PTH, thus allowing for a broader view of calcium homeostasis. A weakness of this study is that the follow-up was conducted within the community setting where certain tests are less accessible; however, since the hypercalcemia was mild and the patient was asymptomatic, there was no need for further evaluation in a hospital.

## Conclusions

In conclusion, we present a case of atorvastatin exacerbating hypercalcemia in the context of hyperparathyroidism. The observed elevations and depressions in calcium levels during the two administrations and cessations of atorvastatin offer evidence of a possible causative effect. The mechanism may be associated with altered vitamin D metabolism, as indicated by the parallel trends of calcium and vitamin D. Future research should explore additional potential mechanisms and interplay between atorvastatin, calcium, vitamin D, and PTH. Additionally, this case highlights the importance of monitoring calcium levels in patients on statins, especially those with existing disorders of calcium homeostasis.
